# Comparison of Newtonian and Special-Relativistic Trajectories with the General-Relativistic Trajectory for a Low-Speed Weak-Gravity System

**DOI:** 10.1371/journal.pone.0034720

**Published:** 2012-04-19

**Authors:** Shiuan-Ni Liang, Boon Leong Lan

**Affiliations:** School of Science, Monash University, Bandar Sunway, Selangor, Malaysia; University of Nottingham, United Kingdom

## Abstract

We show, contrary to expectation, that the trajectory predicted by general-relativistic mechanics for a *low-speed weak-gravity* system is not always well-approximated by the trajectories predicted by special-relativistic and Newtonian mechanics for the same parameters and initial conditions. If the system is dissipative, the breakdown of agreement occurs for chaotic trajectories only. If the system is non-dissipative, the breakdown of agreement occurs for chaotic trajectories and non-chaotic trajectories. The agreement breaks down slowly for non-chaotic trajectories but rapidly for chaotic trajectories. When the predictions are different, general-relativistic mechanics must therefore be used, instead of special-relativistic mechanics (Newtonian mechanics), to correctly study the dynamics of a weak-gravity system (a low-speed weak-gravity system).

## Introduction

For dynamical systems where gravity does not play a dynamical role, it is expected (see, for example, [1]–[3]) that, if the speed of the system is *low* (i.e., much less than the speed of light *c)*, the dynamics predicted by special-relativistic mechanics is always well-approximated by the prediction of Newtonian mechanics for the same parameters and initial conditions. However, in a recent study on a model Hamiltonian system [4], we found, contrary to expectation, that the Newtonian trajectory does not remain close to the special-relativistic trajectory although the particle speed is low – the two trajectories eventually become completely different regardless of whether the trajectories are chaotic or non-chaotic. But the agreement between the Newtonian and special-relativistic trajectories breaks down much faster – exponentially fast – in the chaotic case compared to the non-chaotic case. Similar rapid breakdown of agreement was also found in a model dissipative system [5], [6] and a model scattering system [7] in the chaotic case but no breakdown of agreement was found in the non-chaotic case. The loss of agreement means [6]–[8] that special-relativistic mechanics must be used, instead of the standard practice of using Newtonian mechanics, to correctly study the dynamics of a low-speed system.

For dynamical systems where gravity does play a dynamical role but gravity is *weak* (i.e., gravitational potential ≪*c* ^2^ [9]), it is expected (see, for example, [3], [10], [11]) that the dynamical prediction of general-relativistic mechanics is always well-approximated by the prediction of special-relativistic mechanics for the same parameters and initial conditions. Furthermore, if gravity is *weak* and the speed of the system is *low*, the dynamical prediction of general-relativistic mechanics is expected (see, for example, [3], [10]–[13]) to be always well-approximated by the Newtonian prediction for the same parameters and initial conditions. In this paper, we study a *low-speed weak-gravity* system – the bouncing ball system [14], [15] – to ascertain if these expectations are correct by comparing the Newtonian and special-relativistic trajectories with the general-relativistic trajectory. In a recent paper [16], only the Newtonian and general-relativistic trajectories were compared, with the assumption that, in between impacts with the table, the ball free falls in an exact uniform gravitational field. Here, the gravitational field of the earth is instead modeled as the field due to a uniform sphere – this leads to a different general-relativistic description of the free-fall motion and consequently of the bouncing ball dynamics. Moreover, in the previous paper [16], only inelastic collision between the ball and table was considered; here, both elastic and inelastic collisions are considered. Details of the bouncing ball system and the Newtonian and relativistic trajectory calculations are given next. This is followed by the results and discussion, and concluding remarks on their significance.

## Methods

The bouncing ball system [14], [15] consists of a ball bouncing repeatedly on a table which is oscillating sinusoidally with amplitude *A* and frequency *ω*. The impact between the ball and the table is instantaneous, where the coefficient of restitution *α* (0≤*α*≤1) measures the kinetic energy loss of the ball at each impact: the impact is elastic if *α* = 1, inelastic if *α*<1. The table is not affected by the impact because the table's mass is much larger than the ball's mass. In between impacts, the ball undergoes free-fall motion due to the gravitational field of the earth, which is assumed to be a uniform sphere.

In the Newtonian framework, the dynamics of the bouncing ball is described by the two-dimensional map derived by Tufillaro and co-workers [14], [15]. Following [14], [15], we derive the special-relativistic map and general-relativistic map in terms of the ball's velocity *v* and the table's phase *θ* just after each impact. The table's phase is given by (*ωt*+*θ*_0_) modulus 2*π*. We will refer to the table's phase just after each impact as the impact phase. Our derivations (see Text S1 and S2) of the relativistic maps for the bouncing ball follow the same steps as the derivation [14], [15] of the Newtonian map.

In the Newtonian framework, the dynamics of the bouncing ball is [14], [15] described by the impact-phase map
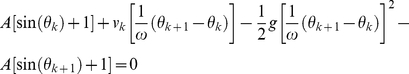
(1)and the velocity map
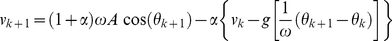
(2)where 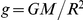, *M* and *R* are respectively the mass and radius of the earth, and *G* is the gravitational constant.

In the special-relativistic framework, the impact-phase map is
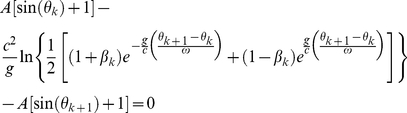
(3)where 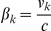. The velocity map is
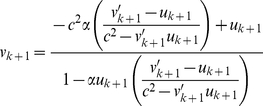
(4)where 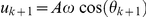 is the table's velocity just after the (*k*+1)th impact, and
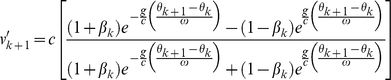
is the ball's velocity just before the (*k*+1)th impact.

In the general-relativistic framework, the impact-phase map is
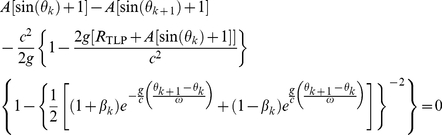
(5)where the constant *R*_TLP_ is the distance between the table's lowest position and the center of the earth. The velocity map is also given by Eq. (4).

The general-relativistic map [Eqs. (5) and (4)] is approximately the same as the special-relativistic map [Eqs. (3) and (4)] if gravity is *weak* [2*g*(*R*_TLP_+*y*)/*c* ^2^≪1 and 2*g*(*R*_TLP_+*y*_0_)/*c* ^2^≪1], where *y* is the ball's position relative to *R*_TLP_. And the general-relativistic map is approximately the same as the Newtonian map [Eqs. (1) and (2)] if the ball's speed and table's speed are *low* [*v*/*c*≪1, *v*_0_/*c*≪1, *g*(*t*−*t*_0_)/*c*≪1 and *u*/*c*≪1] and gravity is *weak*. Furthermore, the special-relativistic map is approximately the same as the Newtonian map if the ball's speed and table's speed are *low*.

To time-evolve the Newtonian and relativistic trajectories, the impact-phase maps Eq. (1), Eq. (3) and Eq. (5), which are implicit algebraic equations for *θ_k_*_+1_, must be solved numerically by finding the zero of the function on the left side of the equation given *θ_k_* and *v_k_*. We used Brent's method for this purpose. First, each trajectory is calculated in quadruple precision (35 significant figures) with a tolerance of 10^−30^ for the zeros. The trajectory is then recalculated in quadruple precision but using a smaller tolerance of 10^−32^ for the zeros. Finally, the accuracy of the trajectory is determined by the standard method [17] of comparing the less-accurate calculation (10^−30^-tolerance) with the more-accurate calculation (10^−32^-tolerance). For example, if the Newtonian velocity is 7.123456789… from the 10^−30^-tolerance calculation and 7.123456799… from the 10^−32^-tolerance calculation, then it is accurate to 8 significant figures, i.e., 7.1234567. We used *g* = 981 cm/s^2^, *c* = 3×10^10^ cm/s, and *R*_TLP_ = 6.4×10^8^ cm (mean radius of the Earth).

The trajectory generated by each of the three maps can be chaotic. A trajectory is defined [18] as chaotic if it exhibits sensitive dependence on initial conditions, that is, the distance between the trajectory and another initially-nearby trajectory from the same theory grows, on average, exponentially for a short time, where the exponential growth constant is not exactly equal to but close to the Lyapunov exponent which is a long-time asymptotic quantity. To determine if a trajectory is chaotic, we inspect the trajectory in phase space, check for sensitivity of the trajectory to initial conditions and calculate [18] the largest Lyapunov exponent to see if it is positive.

In the following results section, instead of reporting the impact phase *θ*, i.e., the table's phase just after each impact, we report the ball's position (which is also the table's position) *y* = *A*[sin(*θ*)+1] just after each impact, together with the ball's velocity *v* just after each impact, when comparing the predictions of the three theories.

## Results

Three examples are presented and discussed to illustrate the general results. In all three examples, the ball's speed and table's speed remained *low* (about 10^−10^ *c*), and gravity is *weak* (2*g*(*R*_TLP_+*y*) is about 10^−9^ *c* ^2^).

In the first two examples, the system is dissipative with *α* = 0.5. In both examples, the initial conditions are 0.02022 cm for the ball's position and 8.17001 cm/s for the ball's velocity. The table's frequency (*ω*/2*π*) is 60 Hz, but the table's amplitude *A* is slightly different: 0.0102 cm in the first example, 0.012 cm in the second example.

**Figure 1:**
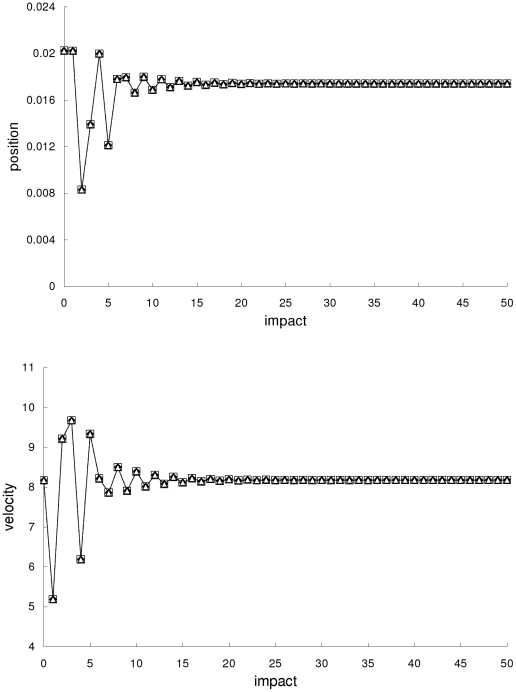
Comparison of trajectories for the first example. Comparison of the Newtonian (squares), special-relativistic (diamonds) and general-relativistic (triangles) positions (top plot) and velocities (bottom plot) for the non-chaotic first example.

In the first example, the Newtonian, special-relativistic and general-relativistic trajectories are all non-chaotic. Fig. 1 shows that the three trajectories are close to one another and they converge to period-one fixed-point attractors which are almost identical.

**Figure 2:**
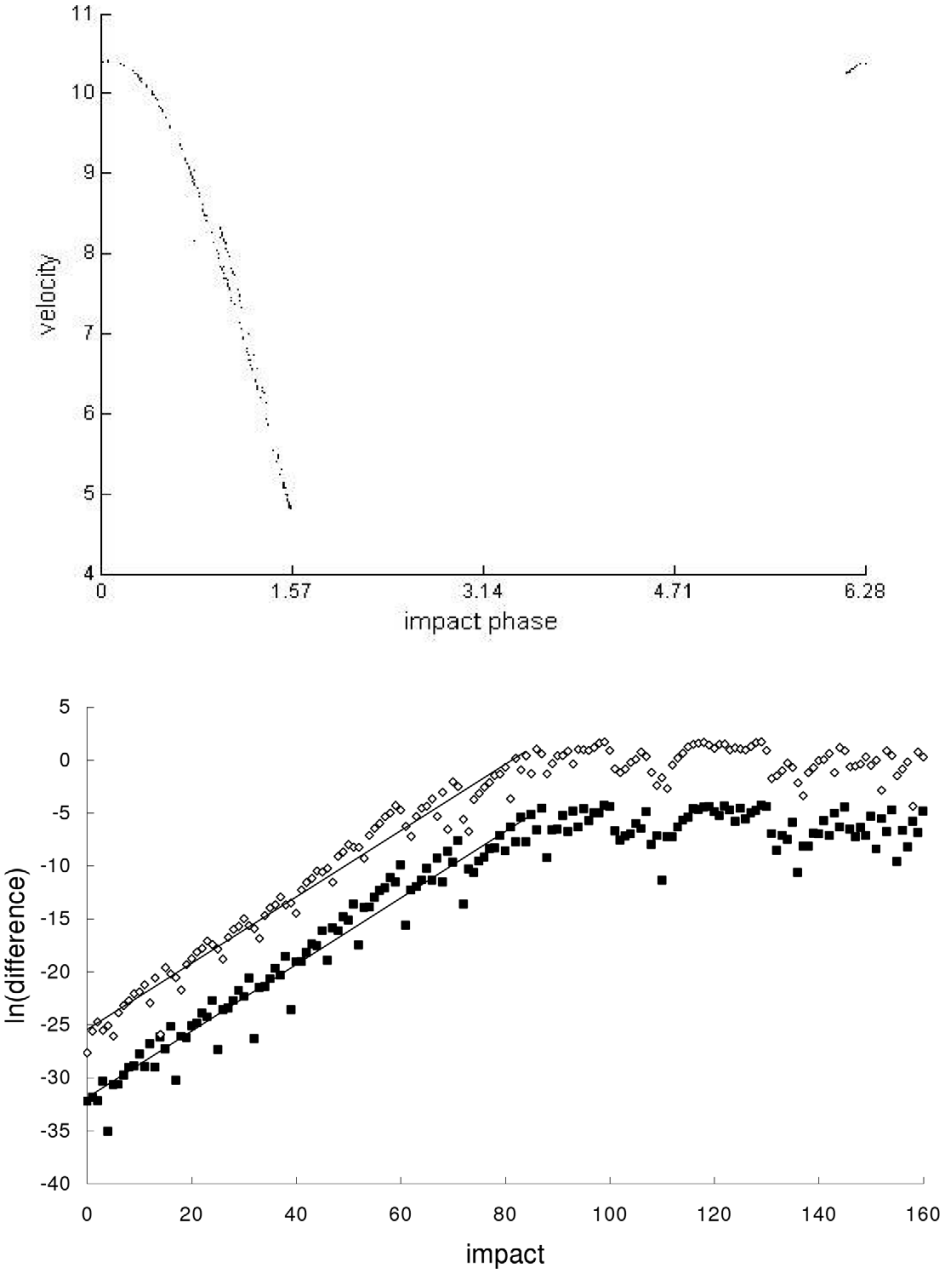
Newtonian trajectory for the second example. Top: Chaotic Newtonian phase-space trajectory, plotted for the first 210 impacts, from the second example. Bottom: Natural-log of the magnitude of the difference [position difference (squares), velocity difference (diamonds)] between the chaotic Newtonian trajectory and another Newtonian trajectory which differed initially by 10^−14^ in position and 10^−12^ in velocity. Straight-line fits up to impact 84 are also plotted.

**Figure 3:**
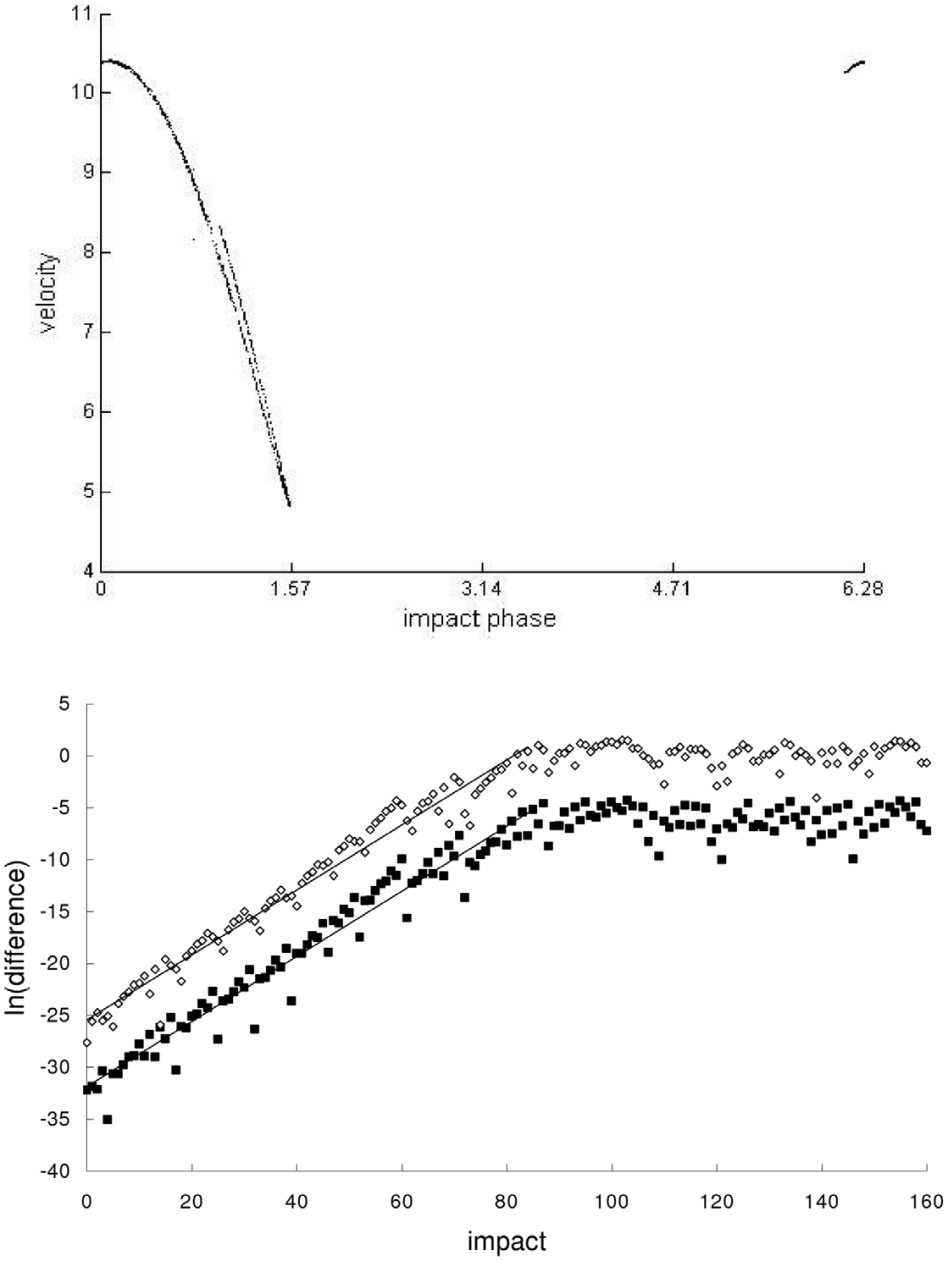
Special-relativistic trajectory for the second example. Top: Chaotic special-relativistic phase-space trajectory, plotted for the first 1000 impacts, from the second example. Bottom: Natural-log of the magnitude of the difference [position difference (squares), velocity difference (diamonds)] between the chaotic special-relativistic trajectory and another special-relativistic trajectory which differed initially by 10^−14^ in position and 10^−12^ in velocity. Straight-line fits up to impact 84 are also plotted.

**Figure 4:**
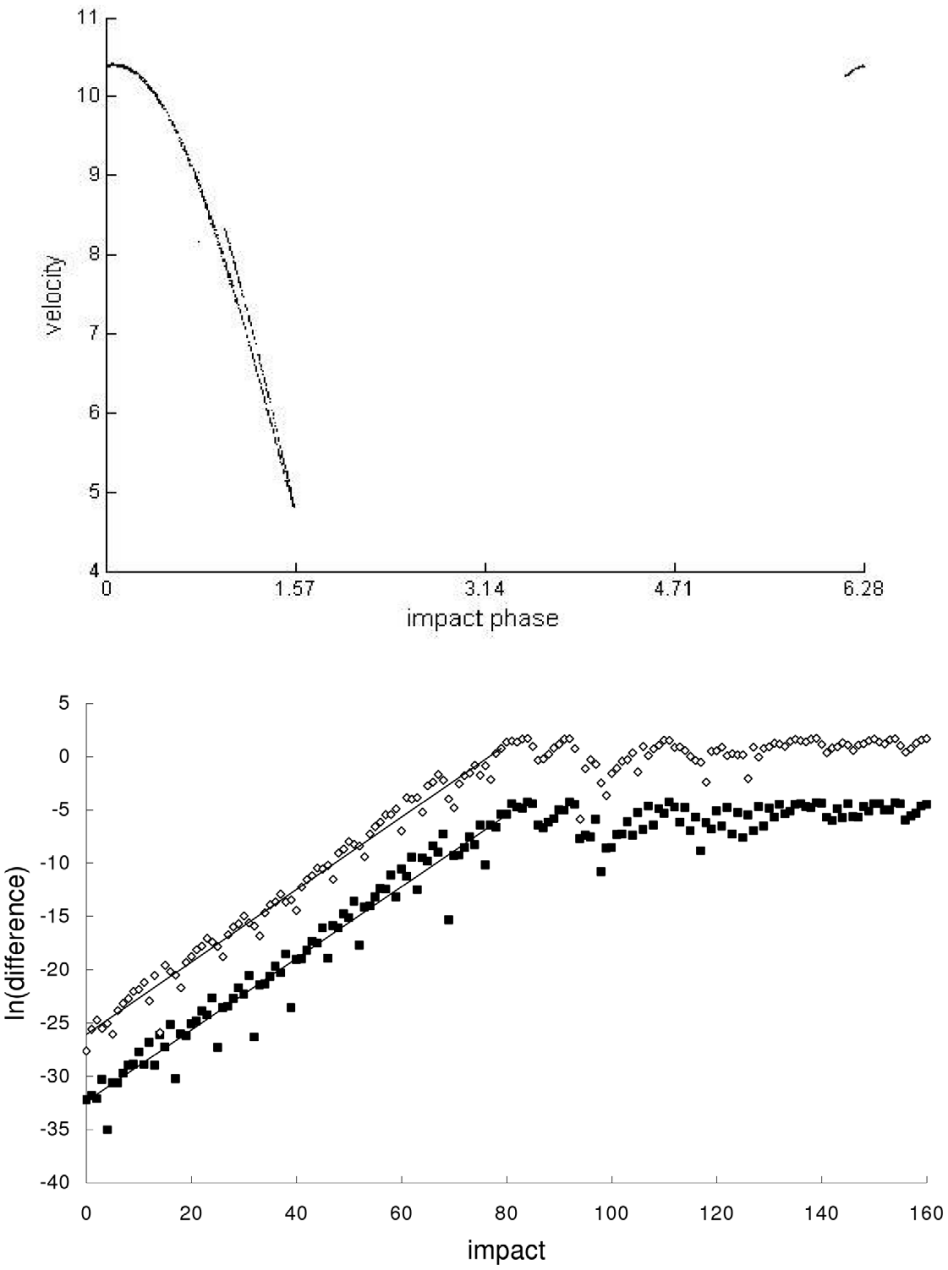
General-relativistic trajectory for the second example. Top: Chaotic general-relativistic phase-space trajectory, plotted for the first 1000 impacts, from the second example. Bottom: Natural-log of the magnitude of the difference [position difference (squares), velocity difference (diamonds)] between the chaotic general-relativistic trajectory and another general-relativistic trajectory which differed initially by 10^−14^ in position and 10^−12^ in velocity. Straight-line fits up to impact 79 are also plotted.

**Figure 5:**
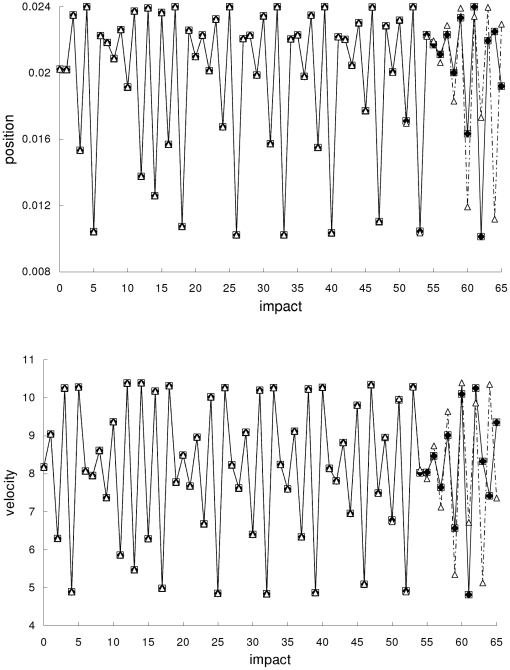
Comparison of trajectories for the second example. Comparison of the Newtonian (squares), special-relativistic (diamonds) and general-relativistic (triangles) positions (top plot) and velocities (bottom plot) for the chaotic second example.

In the second example, the Newtonian, special-relativistic and general-relativistic trajectories, which are plotted in phase space in the top part of Figs. 2, 3 and 4 respectively, are all chaotic as evidenced by the sensitivity to initial conditions (shown in the bottom part of Figs. 2, 3 and 4 respectively) and positive largest Lyapunov exponent of 0.34 for each trajectory. Fig. 5 shows that the agreement between the special-relativistic trajectory and general-relativistic trajectory breaks down very quickly at impact 55, and the agreement between the Newtonian trajectory and general-relativistic trajectory also breaks down at impact 55. The breakdown of agreement between the Newtonian and special-relativistic trajectories (not shown in Fig. 5) occurs later, at impact 95.

**Figure 6:**
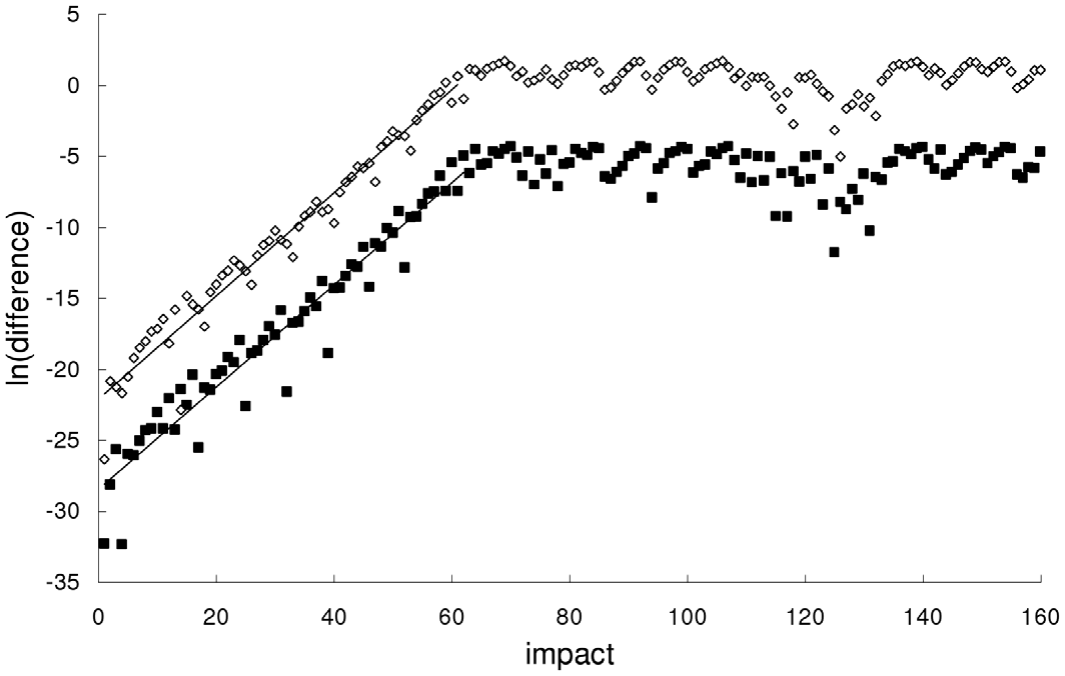
Difference between the special-relativistic and general-relativistic trajectories for the second example. Natural-log of the magnitude of the difference between the special-relativistic and general-relativistic positions (squares) and velocities (diamonds) for the chaotic second example. Straight-line fits up to impact 61 are also plotted.

**Figure 7:**
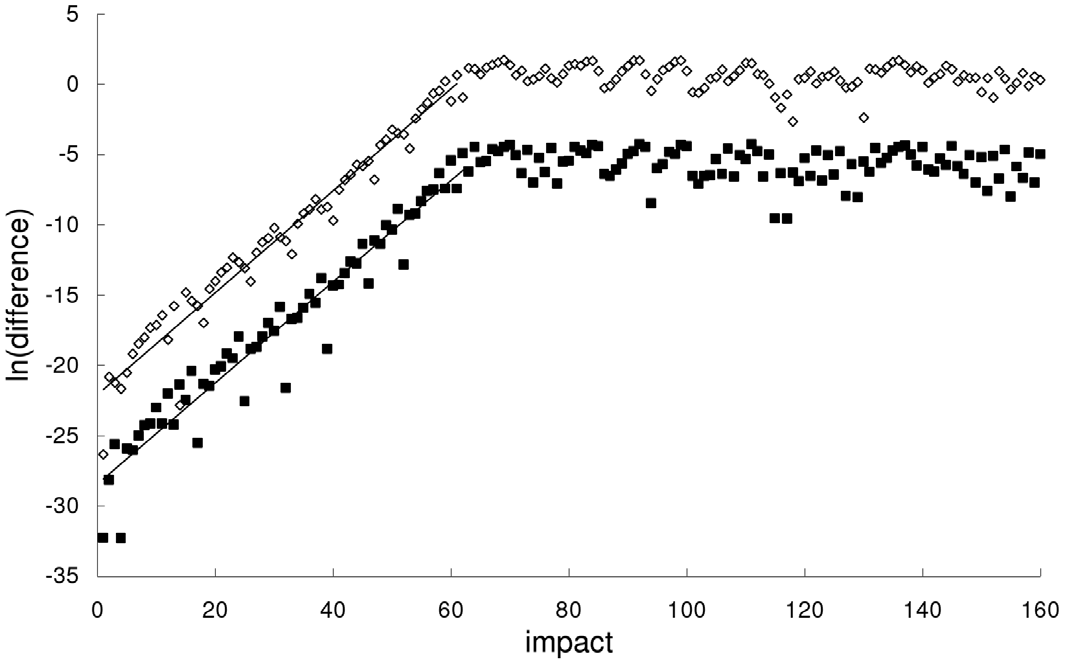
Difference between the Newtonian and general-relativistic trajectories for the second example. Natural-log of the magnitude of the difference between the Newtonian and general-relativistic positions (squares) and velocities (diamonds) for the chaotic second example. Straight-line fits up to impact 61 are also plotted.

Figs. 6 and 7 show, respectively, that the rapid breakdown of agreement between the special-relativistic and general-relativistic trajectories and between the Newtonian and general-relativistic trajectories are due to the, on average, exponential growth – that is, exponential growth with small fluctuations – of the magnitude of the difference between the two trajectories for at least the first 61 impacts:
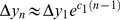
(6)

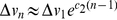
(7)where *n* = 1,2,…. In both cases, the exponential growth constants for the position difference in Eq. (6) and velocity difference in Eq. (7) are close to each other: *c_1_*≈0.360 and *c_2_*≈0.363. This exponential growth constant of about 0.36 is close to (i) the exponential growth constant for the magnitude of the difference (plotted in Figs. 2, 3 and 4) between the chaotic trajectory and another initially-nearby trajectory from the same theory – the growth constants are 0.31, 0.31 and 0.34, respectively, for the Newtonian, special-relativistic and general-relativistic case, where the two nearby trajectories differed initially by 10^−14^ in position and 10^−12^ in velocity, and (ii) the largest Lyapunov exponent of 0.34 for the Newtonian, special-relativistic and general- relativistic chaotic trajectories. We note that the magnitude of the difference between the Newtonian and special-relativistic trajectories also grows exponentially on average, consistent with the results in [4]–[7] for low-speed systems, with growth constants *c_1_*≈0.319 and *c_2_*≈0.320.

**Figure 8:**
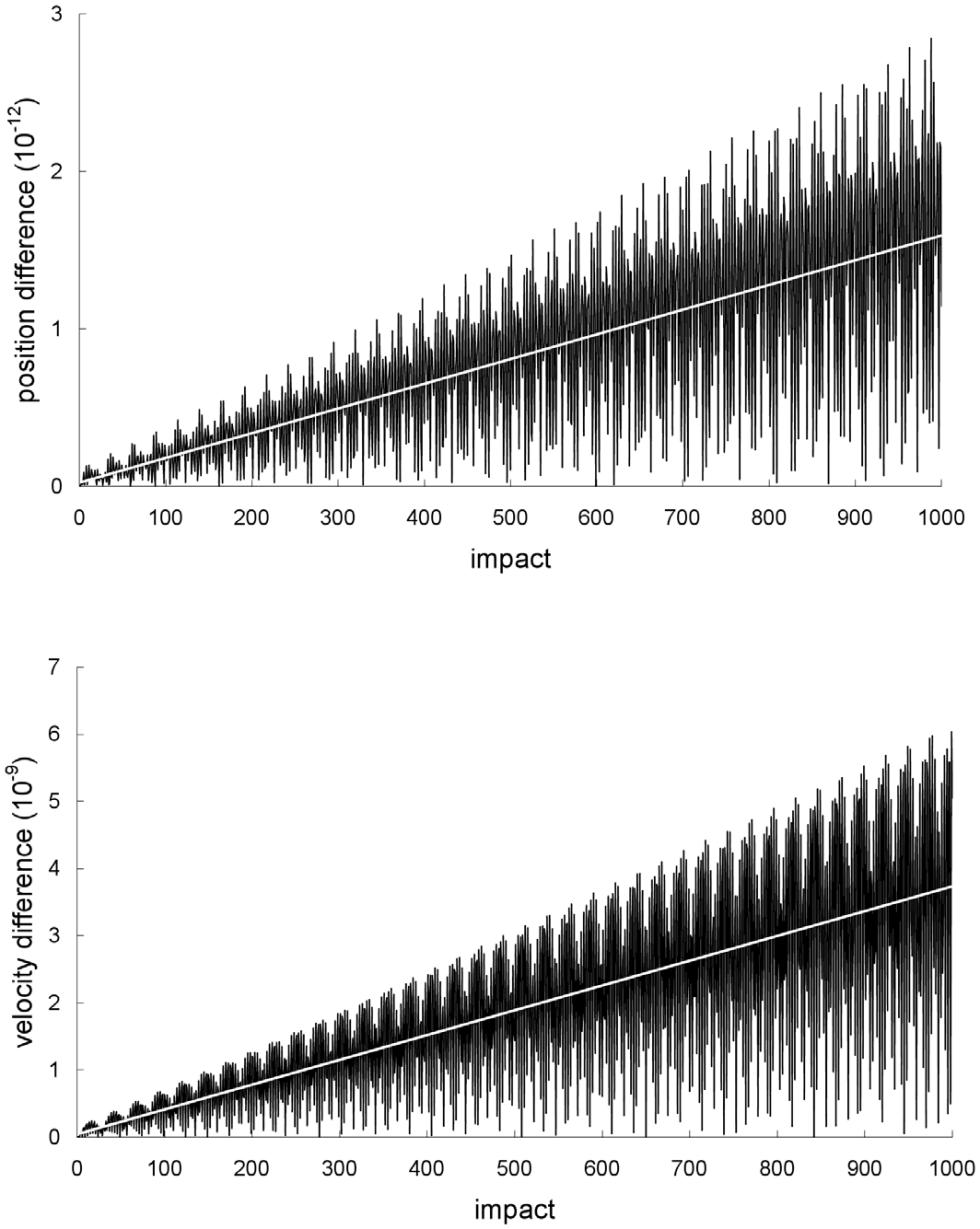
Difference between the Newtonian and general-relativistic trajectories for the third example. Magnitude of the difference between the Newtonian and general-relativistic positions (top plot) and velocities (bottom plot) for the non-chaotic third example. Straight-line fits are also plotted.

**Figure 9:**
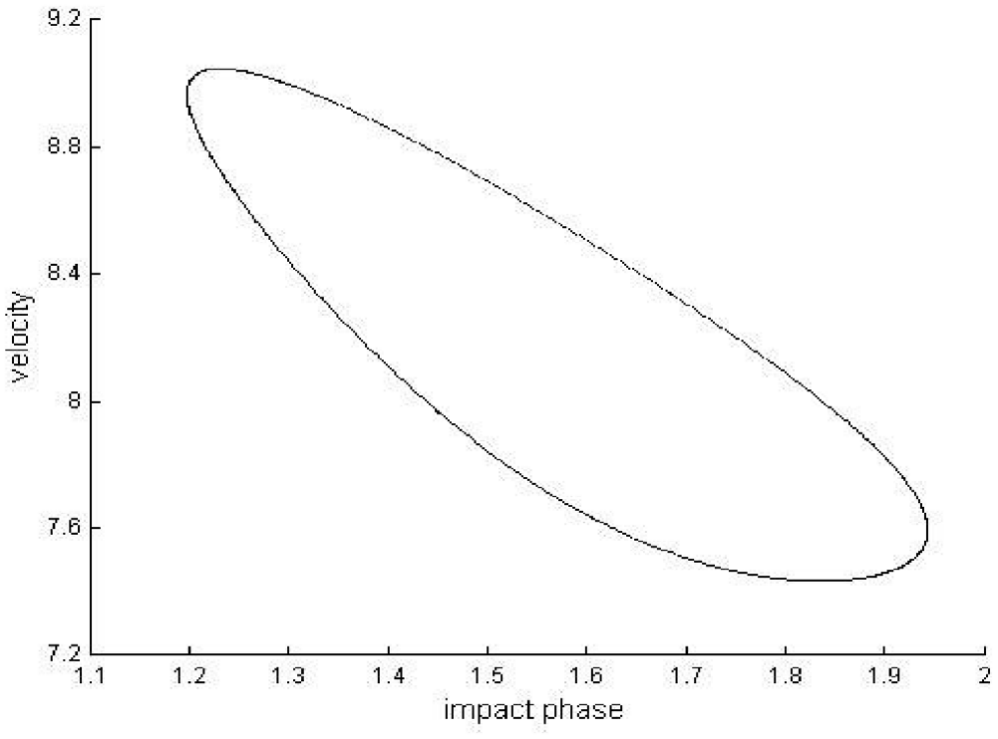
Trajectories for the third example. Quasiperiodic Newtonian, special-relativistic and general-relativistic phase-space trajectories, plotted for the first 1000 impacts, from the non-chaotic third example. The three trajectories are still close to one another at impact 1000 and thus they are indistinguishable in the plot.

In the non-dissipative case, where *α* = 1, the agreement between the special-relativistic and Newtonian chaotic trajectories with the general-relativistic chaotic trajectory also breaks down exponentially fast. The agreement also breaks down for non-chaotic trajectories but it takes a much longer time to occur because the difference between the trajectories only grows linearly. Fig. 8 illustrates this linear growth for the difference between the Newtonian and general-relativistic quasiperiodic trajectories (the trajectories are plotted in phase space in Fig. 9) – in this third example, the table's frequency and amplitude are 60 Hz and 0.005 cm, and the ball's initial position and velocity are 0.00991 cm and 8.17001 cm/s. The linear growth rates of the magnitude of the position difference and velocity difference are 2×10^−15^ cm and 4×10^−12^ cm/s, respectively, per impact. It would thus require 2.5×10^10^ (!) impacts for the magnitude of the velocity difference to grow to 0.1 cm/s. Similar linear growth rates were found for the difference between the special-relativistic and general-relativistic quasiperiodic trajectories in this example (the special-relativistic trajectory is also plotted in Fig. 9).

In general, the breakdown of agreement between the special-relativistic and general-relativistic trajectories for *weak gravity*, and between the Newtonian and general-relativistic trajectories for *low speed* and *weak gravity* can be further understood as follows.

Firstly, rewriting the general-relativistic impact-phase map [Eq. (5)] and taking the natural logarithm on both sides yield
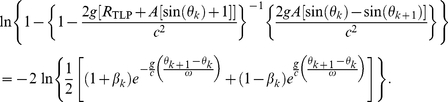
(8)For *weak* gravity, we have 2*g*{*R_TLP_*+*A*[sin(*θ_k_*)+1]}/*c* ^2^≪1 and this implies that the factor {1−2*g*[*R_TLP_*+*A*[sin(*θ_k_*)+1]]/*c* ^2^}^−1^ in the logarithmic function on the left of Eq. (8) is approximately 1. Furthermore, for *weak* gravity, we have 2*g*{*R_TLP_*+*A*[sin(*θ_k_*_+1_)+1]}/*c* ^2^≪1, therefore we can use the expansion ln(1+*x*) = *x*−*x* ^2^/2 for the logarithmic function on the left of Eq. (8) since |*x*|≪1. Consequently, Eq. (8) becomes
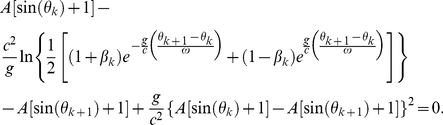
(9)The approximate general-relativistic impact-phase map given by Eq. (9) differs from the special-relativistic impact-phase map [Eq. (3)] by the last term which involves 1/*c* ^2^. The general-relativistic velocity map is exactly the same as the special-relativistic velocity map [Eq. (4)]. The breakdown of agreement between the special-relativistic and general-relativistic trajectories is thus essentially due to the small 1/*c* ^2^ term in Eq. (9).

Secondly, for *weak* gravity, the factor {1−2*g*[*R_TLP_*+*A*[sin(*θ_k_*)+1]]/*c* ^2^} in the general-relativistic impact-phase map [Eq. (5)] is approximately 1. Additionally, for *low* speed, we have *g*(*θ_k_*_+1_−*θ_k_*)/(*cω*)≪1, therefore we can use the expansion *e^x^* = 1+*x*+*x* ^2^/2 for the exponential functions in the term with exponent −2 in Eq. (5) since |*x*|≪1. Furthermore, for *low* speed, we have *v_k_*/*c*≪1, and hence we can expand the resulting (1+*x*)*^−^* ^2^ term as 1−2*x*+3*x* ^2^ since |*x*|≪1. For *low* speed and *weak* gravity, Eq. (5) is thus approximately
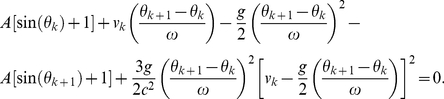
(10)Moreover, for *low* speed, *v′_k_*_+1_/*c*≪1 and *u_k_*_+1_/*c*≪1, and so the general-relativistic velocity map, which is exactly the same as the special-relativistic velocity map [Eq. (4)], is approximately
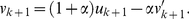
(11)Furthermore, for *low* speed, we can use the expansion *e^x^* = 1+*x*+*x* ^2^/2 for the exponential functions in *v*′*_k_*_+1_ (the expression for *v*′*_k_*_+1_ is given after Eq. 4) since |*x*|≪1, and then expand the resulting (1+*x*)^−1^ term as (1−*x*) since |*x*|≪1. Substituting the resulting approximate expression for *v*′*_k_*_+1_ and *u_k_*_+1_ = *Aω*cos(*θ_k_*_+1_) into Eq. (11) yields
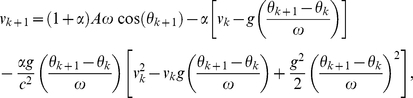
(12)where terms involving 1/*c* ^4^ are omitted since they are very small. The approximate general-relativistic velocity map given by Eq. (12) differs from the Newtonian velocity map [Eq. (2)] by the last term which involves 1/*c* ^2^. Similarly, the approximate general-relativistic impact-phase map given by Eq. (10) differs from the Newtonian impact-phase map [Eq. (1)] by the last term which involves 1/*c* ^2^. The breakdown of agreement between the Newtonian and general-relativistic trajectories is therefore essentially due to the small 1/*c* ^2^ term in Eq. (10) and Eq. (12).

## Discussion

The simplicity of the bouncing ball system allows accurate calculations of the Newtonian, special-relativistic and general-relativistic trajectories for comparison, whereas such accurate calculations would be very difficult to achieve in more complex gravitational systems that can also exhibit chaotic behavior, for example, the three-body problem. Furthermore, the bouncing ball system can be realized experimentally – one realization [15] of the system consists of a steel ball bouncing on a concave lens which is attached to the membrane of a sinusoidally-driven loudspeaker. Because the bouncing ball system is a simple but realistic example of low-speed weak-gravity systems that can exhibit chaotic and non-chaotic behavior – i.e., a prototypical system – the breakdown of agreement of the special-relativistic and Newtonian trajectories with the general-relativistic trajectory should also occur in other low-speed weak-gravity systems.

The breakdown of agreement of the special-relativistic and Newtonian trajectories with the general-relativistic trajectory for a low-speed weak-gravity system has two important implications. First, general-relativistic mechanics must be used, instead of special-relativistic mechanics, to correctly study the dynamics of a weak-gravity system. Second, general-relativistic mechanics must be used, instead of the standard practice (see, for example, [19]) of using Newtonian mechanics, to correctly study the dynamics of a low-speed weak-gravity system. These paradigm shifts may well lead to new understandings and discoveries for low-speed weak-gravity systems.

## Supporting Information

Text S1 **Derivation of the special-relativistic and general-relativistic maps.**
(DOC)

Text S2 **Newtonian and relativistic free-fall motion.**
(DOC)
