# Supplementary material for: Comparison of Newtonian and Special-Relativistic Trajectories with the General-Relativistic Trajectory for a Low-Speed Weak-Gravity System
Source: PLoS One. 2012 Apr 19;7(4):e34720. doi: 10.1371/journal.pone.0034720 (PMC3334942; doi:10.1371/journal.pone.0034720)
Supplement: Text S1 — Derivation of the special-relativistic and general-relativistic maps. (DOC) [file pone.0034720.s001.doc]

**Text S1. Derivation of the special-relativistic and general-relativistic maps.**

Following [1,2], the earth is assumed to be a uniform sphere of radius *R*. Furthermore, in between impacts with the table, the ball, which is initially close to the earth’s surface (*r*0  *R*), undergoes free-fall motion along the radial direction where the distance it travels |*r* - *r*­0­| is assumed small compared to its initial position *r*0 (|*r* - *r*0|/*r*0 <<1). The relativistic position and velocity of the ball between impacts (which are derived in Text S2) are needed in the derivations of the relativistic maps. In the derivations, it is convenient to transform the position *r* of the ball, which is measured relative to the center of the earth, to *y*: *y* = *r* - *R*TLP, where *R*TLP is the distance from the center of the earth to the table’s lowest position. The table’s position is measured relative to *R*TLP.

In between the *k*th and (*k*+1)th impacts, the ball moves with initial velocity *vk* and position *yk* just after the *k*th impact. The ball’s initial position *yk* is the same as the table’s position just after the *k*th impact, where is the table’s phase and *tk* is the time just after the *k*th impact. In the general-relativistic framework, the ball’s position at time *t* after the *k*th impact is [based on Eq. (B17) in Text S2]

,

(A1)

and the ball’s velocity at time *t* after the *k*th impact is [based on Eq. (B9) in Text S2]

. (A2)

Setting the difference between the ball’s position *y*(*t*) [Eq. (A1)] and table’s position at the (*k*+1)th impact to zero yields the impact-phase mapEq. (5).

Since the collision between the ball and table is inelastic, $\bar{v}'_{k+1}$ and $\bar{v}_{k+1}$, which are respectively the ball’s velocity just before and just after the (*k*+1)th impact in the *table’s* reference frame, are related through

(A3)

where *α* is the coefficient of restitution. The ball’s velocity just before and just after the (*k*+1)th impact in the *ground’s* reference frame are respectively

and , (A4)

where $u_{k+1}$ is the table’s velocity at the (*k*+1)th impact in the ground’s reference frame. Solving for $\bar{v}'_{k+1}$ and $\bar{v}_{k+1}$ from Eq. (A4) and substituting into Eq. (A3) yields the velocity map Eq. (4). The expression for $v'_{k+1}$ is obtained by substituting into Eq. (A2).

In the derivation of the special-relativistic map, Eq. (A1) is replaced by the special-relativistic position of the ball between the *k*th and (*k*+1)th impacts based on the special-relativistic Eq. (B8) in Text S2. In the derivation [1,2] of the Newtonian map, the Newtonian Eqs. (B5) and (B6) in Text S2 were utilized to obtain the position and velocity of the ball between the *k*th and (*k*+1)th impacts, and Eq. (A4) is used without the terms involving *c2*.

**References**

1. Tufillaro NB, Mello TM, Choi YM, Albano AM (1986) Period doubling boundaries of a bouncing ball. J. Physique 47: 1477-1482.
2. Tufillaro NB, Abbott T, Reilly J (1992) An experimental approach to nonlinear dynamics and chaos. California: Addison-Wesley.
