# Supplementary material for: Comparison of Newtonian and Special-Relativistic Trajectories with the General-Relativistic Trajectory for a Low-Speed Weak-Gravity System
Source: PLoS One. 2012 Apr 19;7(4):e34720. doi: 10.1371/journal.pone.0034720 (PMC3334942; doi:10.1371/journal.pone.0034720)
Supplement: Text S2 — Newtonian and relativistic free-fall motion. (DOC) [file pone.0034720.s002.doc]

**Text S2. Newtonian and relativistic free-fall motion.**

Here we consider the radial motion of a particle of mass *m* due to the gravitational field of a uniform sphere of mass *M* and radius *R*.

In the Newtonian framework, the change in gravitational potential energy of the particle from an initial position *r*0 to a final position *r* is given by

. (B1)

If the distance travelled by the particle is small compared to *r*0, that is, |*r* - *r*­0­|/*r*0 <<1, then 1/*r* is essentially given by

, (B2)

since higher-order terms involving (*r* - *r*­0­)/*r*0 are negligible. If the particle is initially near the surface of the sphere, that is, *r*0  *R*, then

*GM*/*r*02  *GM*/*R*2 = *g*. (B3)

Substituting Eqs. (B2) and (B3) into Eq. (B1) reduces Eq. (B1) to approximately the change in gravitational potential energy of a particle in a uniform gravitational field

Δ*U*  *mgr*- *mgr*0.. (B4)

The Newtonian position and velocity of the particle at time *t* are therefore given by the well-known equations:

$r-r_{0}=v_{0}\left(t-t_{0}\right)-\frac{1}{2}g\left(t-t_{0}\right)^{2}$, (B5)

$v=v_{0}-g\left(t-t_{0}\right)$. (B6)

In the special-relativistic framework, if |*r* - *r*­0­|/*r*0 <<1 and *r*0  *R*, Eqs. (B2) and (B3) reduce the change in gravitational potential energy of the particle to

$\Delta U=-\frac{GMm}{\sqrt{1-\left(v/c\right)^{2}}}\left[\frac{1}{r}-\frac{1}{r_{0}}\right]\approx\frac{mgr-mgr_{0}}{\sqrt{1-\left(v/c\right)^{2}}}$. (B7)

Solution of the special-relativistic equation of motion with the force derived from the gravitational potential energy *U*(*r*) in Eq. (B7) yields [1-3]

, (B8)

(B9)

for the position and velocity of the particle at time *t*.

In the general-relativistic framework, the gravitational field outside the uniform sphere is described by the Schwarzschild metric [4] in terms of the Schwarzschild coordinates (*ct*, *r*, *θ*, *φ*)

, (B10)

where *ds* is the interval between neighboring events, *τ* is the proper time, and *r*s = 2*GM*/*c*2 is the Schwarzschild radius. For purely radial motion [3,5] along the line *φ* =constant in the equatorial plane *θ* = *π*/2, the metric Eq. (B10) is simplified, with *dφ* = *dθ* = 0, to

(B11)

and the geodesic equations are reduced to

, (B12)

. (B13)

The local velocity [4,6] of the particle, measured by a local observer who is at rest at a particular Schwarzschild radial coordinate and is next to the particle, is

. (B14)

The integral of Eq. (B12), which is given by

, (B15)

where *k* is a constant, and the integral of Eq. (B13), which is given by Eq. (B11), together with the initial condition *v* = *v*0 at *r* = *r*0, lead to the following expression for *dr*/*dt*:

. (B16)

If |*r* - *r*­0­|/*r*0 <<1 and *r*0  *R*, substituting Eqs. (B2) and (B3) into Eq. (B16) and integrating it with initial condition *r* = *r*0 at *t* = *t*0 yields the general-relativistic position of the particle at time *t*

$r-r_{0}=-\frac{c^{2}}{2g}\left(1-\frac{2gr_{0}}{c^{2}}\right)\left\{1-\left\{\frac{1}{2}\left[\left(1+\frac{v_{0}}{c}\right)e^{-\frac{g\left(t-t_{0}\right)}{c}}+\left(1-\frac{v_{0}}{c}\right)e^{\frac{g\left(t-t_{0}\right)}{c}}\right]\right\}^{-2}\right\}$. (B17)

In the limit of weak gravity (2*gr*/*c*2<<1 and 2*gr*0/*c*2<<1), Eq. (B17) reduces to the special-relativistic Eq. (B8). In the limit of weak gravity and low speed (*v*/*c*<<1, *v*0/*c*<<1 and *g*(*t* – *t*0)/*c*<<1), Eq. (B17) reduces to the Newtonian Eq. (B5).

Substituting Eqs. (B14), (B2), (B3) and (B17) sequentially into Eq. (B16) yields the general-relativistic velocity of the particle at time *t*, which is the same as the special-relativistic Eq. (B9). In the limit of low speed, Eq. (B9) reduces to the Newtonian Eq. (B6).

**References**

1. Lapidus IR (1972) The falling body problem in general relativity. Am. J. Phys. 40: 1509-1510.
2. Lapidus IR (1972) Motion of a relativistic particle acted upon by a constant force and a uniform gravitational field. Am. J. Phys. 40: 984-988.
3. Srinivasa Rao KN (1966) The motion of a falling particle in a Schwarzschild field.Ann. Inst. Henri Poincare, Sect. A 5: 227-233.
4. Landau LD, Lifshitz EM (1975) The classical theory of fields. Oxford: Pergamon Press.
5. Srinivasa Rao KN, Gopala Rao AV (1974) Falling body in the theories of gravitation. J. Phys. A 7**:** 485-488.
6. Zel'dovich YaB, Novikov ID (1996) Relativistic astrophysics vol. 1: Stars and relativity. New York: Dover Publications.
